# Definitive Radiotherapy for Older Patients with Prostate Cancer: Experience of a Medical Center in Taiwan

**DOI:** 10.1038/s41598-017-13119-3

**Published:** 2017-10-24

**Authors:** Yuan-Hung Wu, Wan-Chin Yang, Yu-Wen Hu, Chuen-Mei Hsieh, Kai-Lin Yang, I.-Chun Lai, Chen-Xiong Hsu, Ti-Hao Wang, Tzu-Yu Lai, Kuan-Ting Chen, Yu-Mei Kang, Yu-Ming Liu

**Affiliations:** 1 Division of Radiation Oncology, Department of Oncology, Taipei Veterans General Hospital, Taipei, Taiwan; 2 School of Medicine, National Yang-Ming University, Taipei, Taiwan; 3 Institute of Public Health, National Yang-Ming University, Taipei, Taiwan; 4 Department of Biomedical Imaging and Radiological Sciences, National Yang-Ming University, Taipei, Taiwan; 5 School of Medicine, Fu Jen Catholic University, New Taipei City, Taiwan; 6 Department of Radiation Therapy and Oncology, Shin Kong Wu Ho-Su Memorial Hospital, Taipei, Taiwan; 7 Department of Radiation Oncology, Far Eastern Memorial Hospital, New Taipei City, Taiwan; 8 Department of Radiation Oncology, China Medical University Hospital, Taichung, Taiwan

## Abstract

Whether age predicts treatment outcome of prostate cancer remains controversial. With the aging of the world population, properly understanding the effect of age may facilitate both treatment decision-making and defining the natural history of prostate cancer. Consecutive 581 patients with locally-confined adenocarcinoma of the prostate who received radical definitive radiotherapy(RT) (76–78 Gy) between 2004 and 2015 at a medical center in Taiwan were reviewed retrospectively. Median age was 78 years. The median follow-up was 66 months. The 5-year biochemical failure-free survival(BFFS), distant metastasis-free survival(DMFS), disease-specific survival(DSS), and overall survival(OS) rates were 84.9%, 93.8%, 97.8%, and 86.6%, respectively, for all patients. Comparing those above and below the age of 80, no difference in 5-year BFFS, DMFS, or DSS was found. Multivariate Cox regression analysis showed that tumor stage, Gleason score, initial PSA, and latency before RT were significant risk factors of BFFS. The latency before RT was significantly longer in the older group than in the under 80 group. Delay to start RT might explain the previous finding of inferior disease control in older patients in other studies. With the exception of OS, no other differences in outcomes or toxicities were observed in older patients.

## Introduction

Prostate cancer develops mainly in older men^1,2^. With the increasing life expectancy worldwide^3^, the number of older patients with prostate cancer is expected to rise. Compared with younger patients, older patients are more likely to have an aggressive form of the disease at diagnosis, but more often receive conservative treatment, including watchful waiting and primary androgen-deprivation therapy (ADT)^4,5^. A Surveillance, Epidemiology, and End Results (SEER) study of Medicare-linked data from 2004 to 2005 indicated that among 75-year-old or older prostate cancer patients, even in the high-risk group, up to 40.9% received ADT only while 13.6% received only active surveillance or observation^6^. Recent randomized clinical trials investigated whether radiation therapy(RT) results in better outcome when added to ADT^7,8^. However, patients aged 80 or more were excluded in these studies. Some retrospective studies addressed this issue in patients ≥75 years of age^4,9^. However, only one SEER-Medicare study has evaluated patients 80 years of age or older^10^. Whether age predicts treatment outcome remains controversial. Some studies have reported that older patients have worse biochemical control and cancer-specific survival after either radical prostatectomy or RT^11–13^, while others showed no significant differences after including adequate controls for disease risk and treatment category^4,14^. Currently, little is known about prostate cancer treatment results in patients 80 years of age or older. Because the most appropriate treatment strategy for prostate cancer in this age group has become an important issue, in this study, we compared treatment outcome and toxicities in patients ≥80 years old receiving definitive RT at our institution, to treatment outcome and toxicity data from younger patients.

## Materials and Methods

### Patient selection

Our institution was established as a dedicated medical care facility for veterans, serving the oldest male patient population of all of the medical centers in Taiwan. We started intensity-modulated radiation therapy (IMRT) in 2001. After 2 years, IMRT was fully adopted into the treatment of prostate cancer. We conducted a retrospective review of the medical records of patients who received IMRT targeting the prostate in our institution from January 2004 through December 2015. Inclusion criteria were a pathologically confirmed adenocarcinoma of the prostate and administration of definitive IMRT, with or without ADT. Exclusion criteria were radical prostatectomy, the presence of pathological components other than adenocarcinoma, distant metastasis or regional lymphadenopathy noted before RT, a prescribed dose less than 76 Gy, and not completion of the RT course. The eligible patient list was updated annually by the national cancer registry^15^. Taiwan cancer registry has been operating since 1979. It could be assumed that all relevant patients meeting those inclusion criteria during the study period were included. This study was approved by institutional review board of Taipei Veterans General Hospital (2014-11-006AC) and conducted in accordance to the Declaration of Helsinki. Permission to waive the informed consent was obtained from the institutional review board for this observational retrospective study.

### Tumor staging

Digital rectal examination (DRE) was performed for primary T staging. All patients had transrectal sonography, computed tomography (CT), and whole body bone scan before RT. Final tumor staging depended on integration of DRE and medical imaging within 3 months before RT.

### Risk group

Risk of recurrence was classified into three levels defined by National Comprehensive Cancer Network (NCCN) guidelines^16^. Pretreatment serum prostate specific antigen (PSA) level was assessed within 3 months before RT. Patients were classified as low risk if they had clinical T1 to T2a tumors, a Gleason score ≤ 6, and a pretreatment PSA level <10 ng/ml. Patients with stage T3 to T4 tumors, a Gleason score ≥8, or a pretreatment PSA level >20 ng/ml were classified as having high-risk disease. Other patients, meeting all the following conditions: a stage T2b-T2c tumor, a Gleason score of 7, and a pretreatment PSA level 10–20 ng/ml, were classified as having intermediate-risk disease. For patients with more than one pretreatment PSA level and/or repeated biopsy, the highest risk group classification was chosen.

### Definitions of target volumes and critical structures

Indications for seminal vesicle irradiation included positive biopsy, disease invasion by medical imaging, and a calculated risk of >5% based on Roach’s formula^17^. Indications for whole pelvis RT (WPRT) included seminal vesicle involvement identified by medical imaging, a calculated risk of lymph node involvement that was >15% based on Roach’s formula^18^, and high-risk disease. Of the 371 patients meeting these criteria, 324 (87.3%) received WPRT. We did not prescribe WPRT to 39 (10.5%) patients indicated considering their comorbidities. Eight (2.2%) indicated patients did not receive WPRT due to poor performance status.

Following the International Commission on Radiation Units and Measurements (ICRU) 50 recommendations, clinical target volume (CTV) was delineated on individual axial CT slices in all patients by our radiation oncologist and reviewed by another. For those not receiving WPRT, CTV2 included the prostate and bilateral seminal vesicles. For patients treated with WPRT, the CTV2 consisted of the prostate, bilateral seminal vesicles, and bilateral common iliac, proximal external iliac, hypogastric, and obturator lymph nodes.

CTV1 for patients receiving irradiation of seminal vesicles included the prostate and bilateral seminal vesicles. Otherwise, only prostate and proximal seminal vesicles were included in CTV1.

CTV was expanded 10 mm in superior-inferior, 10 mm in the right-left directions, 7 mm in the anterior direction, and 5 mm in the posterior direction to produce a planning target volume (PTV). The rectum, bladder, and bilateral femoral heads were also delineated for each patient. The rectum was contoured on the simulation CT from anal verge to 10 cm proximal. The peritoneal cavity, excluding the rectum and bladder, below L4–L5 was used to define the small bowel region. The individual loops of bowel were not separately outlined. The dose to the ICRU 62 reference points was reported.

### Treatment planning

Patients received a single fraction per day, five days per week. Forty-eight patients (8.3%) received image-guided RT (IGRT) via Tomotherapy. For the other patients, the position was verified with port films every 1–2 weeks without fiducials implanted. We used sequential boost, instead of simultaneous integrated boost.

We use in-house dose volume constraints as published in 2007^19^. The constraints for rectum were a maximum dose of 78 Gy, <20% of volume under 70 Gy, <30% of volume under 60 Gy, and <40% of volume under 50 Gy. The constraints for bladder were <30% of volume under 70 Gy, and <45% of volume under 60 Gy. The constraints for small bowel region were <20% of volume under 70 Gy, and <30% of volume under 50 Gy.

### Hormonal therapy

Patients with intermediate- or high-risk disease received ADT with diethylstilbestrol, flutamide, bicalutamide, cyproterone, leuprorelin, or triptorelin. Types of ADT were grouped into antiandrogen alone, chemical castration alone, or dual blockade. If a patient received multiple types of ADT, the one used longest was registered. The adjuvant ADT was considered incomplete if patients with intermediate-risk disease received ADT for less than 4 months or those with high-risk disease received ADT for less than 2 years. The 5 patients who received orchidectomy were considered to have received ADT since the day of their operations.

### Follow-up

PSA was checked before and after RT. For the first 3 years after RT, PSA was checked approximately every 3 months. Subsequently, 3–5 years after RT, PSA was checked approximately every 6 months and thereafter it was checked annually. Treatment outcomes and toxicity were recorded on both official electronic medical records and department-maintained electronic spreadsheets during each follow-up clinic visit.

### Endpoints

The primary end point of this study was biochemical failure-free survival (BFFS) after the conclusion of RT with biochemical failure as the only event. The American Society for Radiation Oncology (ASTRO) Phoenix’s definition of biochemical failure was adopted, that is, a rise of ≥2 ng/mL above the nadir PSA after external beam RT, with or without ADT (19). Long-term toxicity was defined as suggested by the Common Terminology Criteria for Adverse Events (CTCAE) version 4.0^20^. Secondary endpoints included overall survival (OS), disease-specific survival (DSS), and distant metastasis-free survival (DMFS). Survival rates were calculated from the last date of RT.

### Statistical analysis

We used IBM SPSS version 22 for statistical analysis. Kaplan-Meier curves were calculated for the survival analyses. Associations among categorical and continuous variables were detected with two-tailed Chi-square tests and a two-tailed Student’s t tests, respectively. Statistical significance between factors was determined using the log-rank test. A p-value less than 0.05 was considered significant. The Cox proportional hazards model was applied to estimate hazard ratios and 95% confidence intervals (CIs).

## Results

### Patient characteristics

From 2004 through 2015, 760 patients with locally-confined adenocarcinoma of the prostate received definitive RT with curative intent in our institution. Of these 760 patients, 4 did not complete the treatment course and were excluded from our analysis. There were 175 patients who received a prescribed dose of less than 76 Gy who also were not analyzed. We included the remaining 581 consecutive patients in the study. The characteristics of the final study population are shown in Table 1.

**Table 1:** Baseline demographic and clinical characteristics.

Characteristic		All	Age <80	Age ≥80	P
N (%)		581	380 (65.4)	201 (34.6)	—
Age at RT, median (range)		78 (54–93)	75 (54–79)	82 (80–93)	—
Hypertension, n (%)		333 (57.3)	202 (53.2)	131 (65.2)	0.006*
DM, n (%)		136 (23.4)	96 (25.3)	40 (19.9)	0.151
Aspirin, n (%)		209 (36.0)	126 (33.2)	83 (41.3)	0.057
Hemorrhoid, n (%)		307 (52.8)	202 (53.2)	105 (52.2)	0.862
Staging	MRI, n (%)	180 (31.0)	143 (37.6)	37 (18.4)	<0.001*
	CT only, n (%)	401 (69.0)	237 (62.4)	164 (81.1)	—
	MRS, n (%)	9 (1.5)	7 (1.8)	2 (1.0)	0.723
Risk	Low, n (%)	64 (11.0)	45 (11.8)	19 (9.5)	0.475
	Intermediate, n (%)	185 (31.8)	116 (30.5)	69 (34.3)	—
	High, n (%)	332 (57.1)	219 (57.6)	113 (56.2)	—
T stage	1a-2a, n (%)	179 (30.8)	118 (31.1)	61 (30.3)	<0.001*
	2b-2c, n (%)	243 (41.8)	137 (36.1)	106 (52.7)	—
	3a-4, n (%)	159 (27.4)	125 (32.9)	34 (16.9)	—
PSA	<10 ng/ml, n (%)	200 (34.4)	132 (34.7)	68 (33.8)	0.975
	10–20 ng/ml, n (%)	180 (31.0)	117 (30.8)	63 (31.3)	—
	≥20 ng/ml, n (%)	201 (34.6)	131 (34.5)	70 (34.8)	—
Gleason	<7, n (%)	215 (37.0)	148 (38.9)	67 (33.3)	0.404
	7, n (%)	177 (30.5)	113 (29.7)	64 (31.8)	—
	8–10, n (%)	189 (32.5)	119 (31.3)	70 (34.8)	—
Pre-RT latency (mos.), mean (range)		5.71 (0–170)	5.2 (0–81)	6.7 (0–170)	0.032*
Dose	76 Gy, n (%)	134 (23.1)	87 (22.9)	47 (23.4)	0.918
	78 Gy, n (%)	447 (76.9)	293 (77.1)	154 (76.6)	—
IGRT, n (%)		48 (8.3)	35 (9.2)	13 (6.5)	0.272
Risk of N1 ≥ 15%, n (%)		371 (63.9)	233 (61.3)	138 (68.7)	0.085
Pelvic irradiation, n (%)		324 (55.8)	228 (60.0)	96 (47.8)	0.005*
ADT	Neoadjuvant, n (%)	387 (66.6)	258 (67.9)	129 (64.2)	0.405
	Concurrent, n (%)	406 (69.9)	267 (70.3)	139 (69.2)	0.777
	Adjuvant, n (%)	341 (58.7)	225 (59.2)	116 (57.7)	0.790
	Ever used, n (%)	471 (81.1)	306 (80.5)	165 (82.1)	0.739
	Incomplete, n (%)	314 (54.0)	199 (52.4)	115 (57.2)	0.294
	Antiandrogen alone, n (%)	316 (54.4)	196 (51.6)	120 (59.7)	0.063
	Chemical castration alone, n (%)	134 (23.1)	97 (25.5)	37 (18.4)	0.062
	Dual blockade, n (%)	7 (1.2)	4 (1.1)	3 (1.5)	0.698
Orchidectomy, n (%)		5 (0.9)	4 (1.1)	1 (0.5)	0.664

RT, radiotherapy; DM, diabetes mellitus; MRI, magnetic resonance imaging; CT, computed tomography; MRS, magnetic resonance spectroscopy; PSA, prostate specific antigen; IGRT, image-guided RT; ADT, androgen deprivation therapy.

The mean age was 76.5 years, with a median of age of 78 years. Hypertension was more common in those 80 years of age or older than in the under 80 group. More patients younger than age 80 received magnetic resonance imaging (MRI) for staging. Magnetic resonance spectroscopy was performed in 9 of the 80 patients who had an MRI. There were fewer patients with T3a to T4 stage tumors in those aged 80 or older. The latency before RT was longer in those aged 80 or older and fewer older patients received WPRT.

### Survival analyses

**Figure 1:**
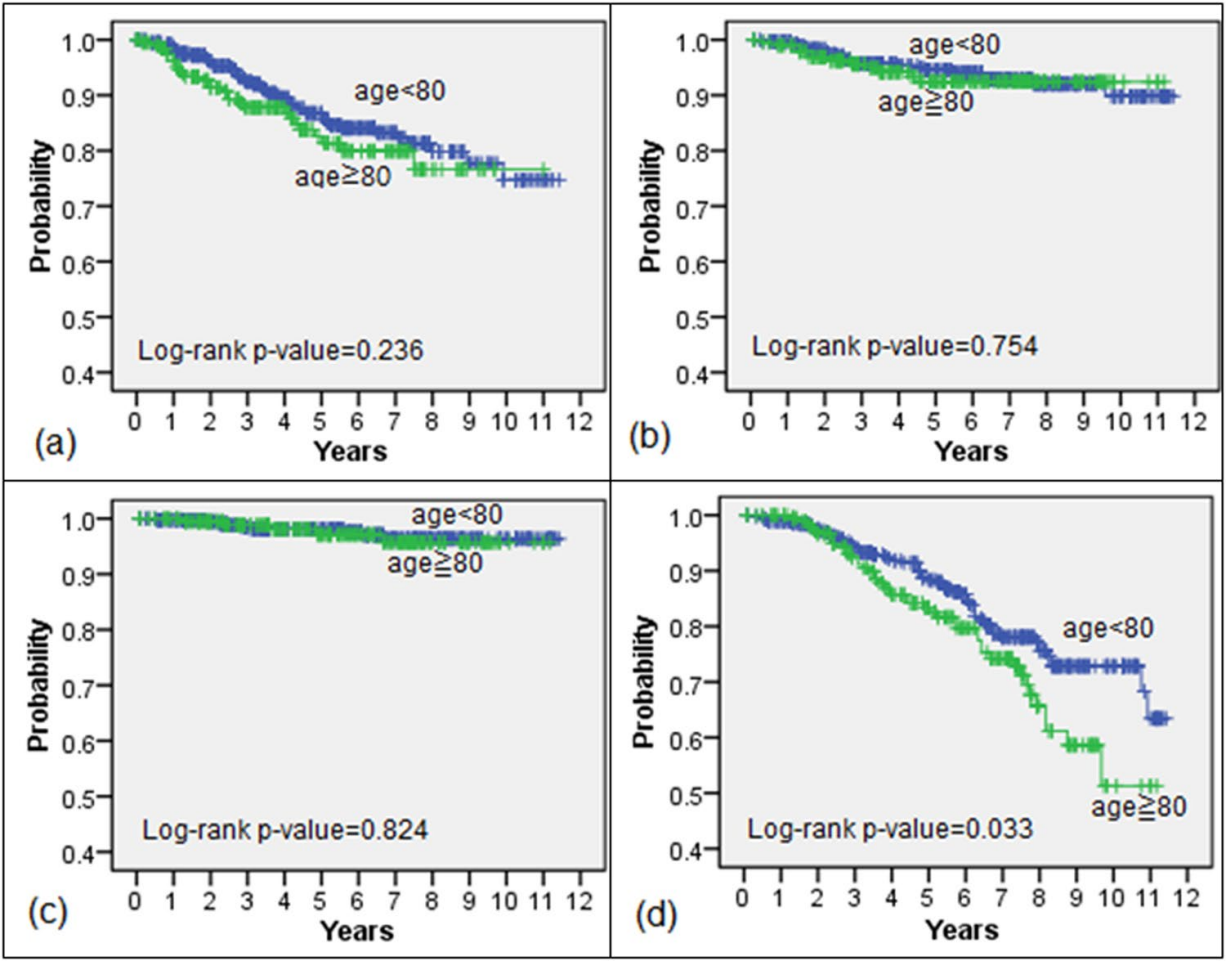
Survival analyses after RT comparing those under age 80 to those age 80 and above. (**a**) biochemical failure-free survival; (**b**) distant metastasis-free survival; (**c**) disease-specific survival; (**d**) overall survival.

The BFFS, DMFS, DSS, and OS of each age group are shown in Fig. 1. The 5-year BFFSs were 86.7% (<80 years old) and 81.4% (≥80 years old). The 5-year DMFSs were 94.5% (<80 years old) and 92.4% (≥80 years old). The 5-year DSSs were 98.1% (<80 years old) and 97.1% (≥80 years old). The 5-year OSs were 88.3% (<80 years old) and 83.4% (≥80 years old). Log-rank test identified no differences in survival, except for OS, between the age groups.

**Figure 2:**
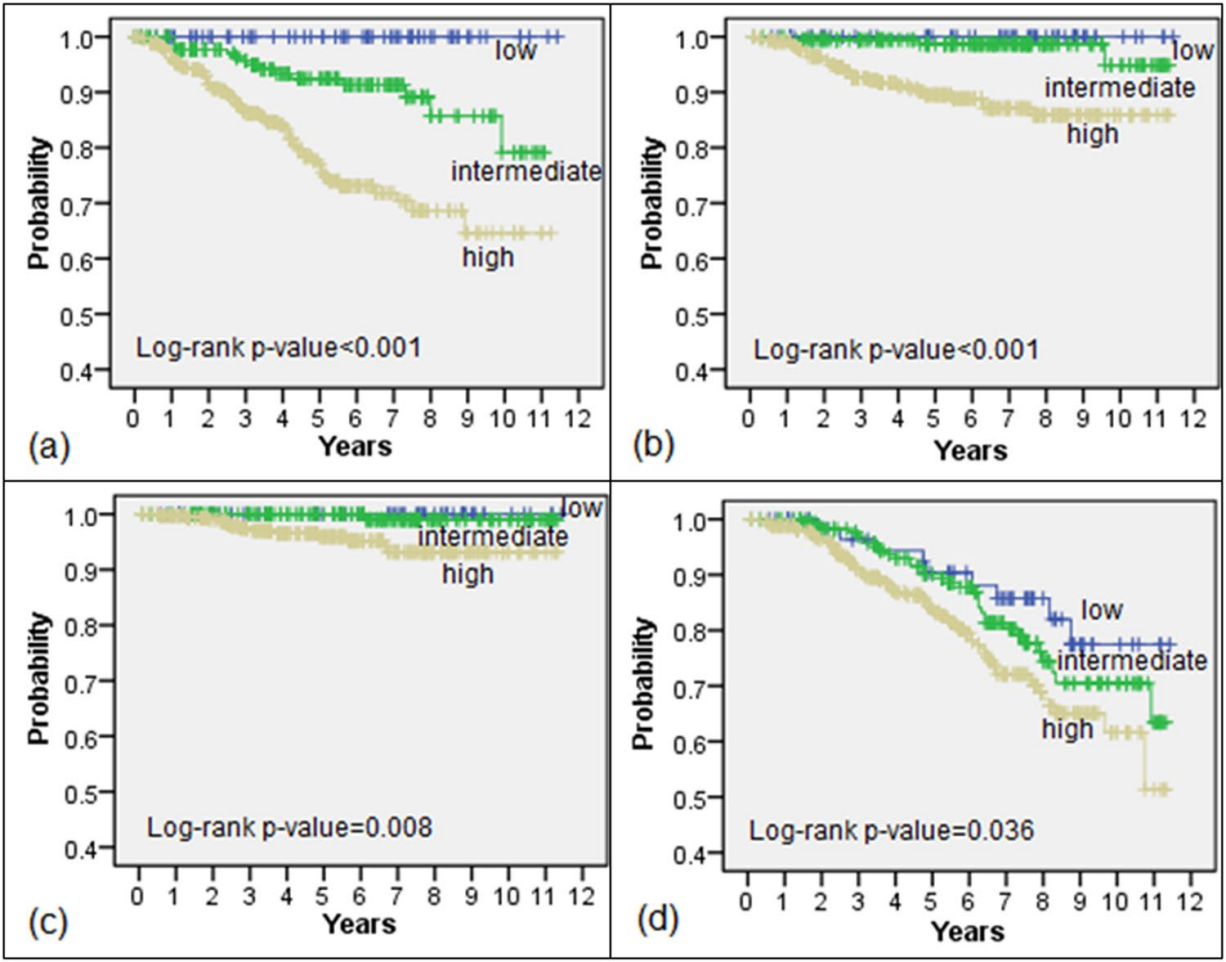
Survival analyses after radiotherapy by risk group. (**a**) biochemical failure-free survival; (**b**) distant metastasis-free survival; (**c**) disease-specific survival; (**d**) overall survival.

The BFFS, DMFS, DSS, and OS data of each risk group are presented in Fig. 2. The 5-year BFFSs of the low, intermediate, and high risk groups were 100%, 92.5%, and 76.7%, respectively. The 5-year DMFSs of these risk groups were 100%, 98.7%, and 89.5%, the 5-year DSSs of these risk groups were 100%, 100%, and 95.9%, and the 5-year OSs of these risk groups were 90.4%, 90.1%, and 83.7%, respectively. All of the survival analysis results differed significantly across risk groups.

Univariate and multivariate Cox regression analyses of BFFS are shown in Table 2. Multivariate Cox regression indicated that latency before RT and risk groups based on tumor stage, Gleason score, and initial PSA were significant prognostic factors.

**Table 2:** Cox proportional hazard model predicting biochemical failure-free survival after intensity-modulated radiation therapy.

Risk factor	Univariate		Multivariate	
	HR (95% CI)	P	HR (95% CI)	P
Age at RT ≥80	1.329 (0.832–2.124)	0.234	1.544 (0.946–2.518)	0.082
T-stage risk group	1.995 (1.465–2.718)	<0.001*	1.447 (1.011–1.881)	0.049*
Gleason score risk group	2.983 (2.154–4.130)	<0.001*	2.805 (1.807–4.354)	<0.001*
Initial PSA risk group	2.195 (1.613–2.986)	<0.001*	1.910 (1.355–2.692)	<0.001*
Incomplete ADT	1.223 (0.772–1.938)	0.339	1.295 (0.736–1.972)	0.358
ADT type^a^	—	<0.001*	—	0.469
No ADT	1	—	1	—
Antiandrogen	0.045 (0.013–0.159)	<0.001*	0.250 (0.041–1.527)	0.133
Chemical castration	0.119 (0.042–0.331)	<0.001*	0.272 (0.059–1.265)	0.097
Dual blockade	0.205 (0.070–0.604)	0.004*	0.291 (0.061–1.396)	0.123
Orchidectomy	0.567 (0.103–3.128)	0.515	0.563 (0.073–4.336)	0.581
Latency before RT (months)	1.023 (1.013–1.034)	<0.001*	1.021 (1.006–1.036)	0.006*
IGRT	1.123 (0.348–3.630)	0.846	0.629 (0.219–2.507)	0.629
Prescribed dose (78 Gy vs. 76 Gy)	1.876 (1.028–3.424)	0.040*	0.501 (0.401–1.563)	0.501
Pelvic irradiation	3.315 (1.949–5.638)	<0.001*	0.152 (0.257–1.235)	0.152

RT, radiotherapy; PSA, prostate specific antigen; ADT, androgen deprivation therapy; IGRT, image-guided RT; ^a^categorical variable.

### Toxicity

RT-related toxicities of the urinary and gastrointestinal (GI) tracts are listed in Table 3. Acute toxicities noted included lower urinary tract symptoms, diarrhea, and anal pain. Late urinary toxicity, including hematuria, noninfective cystitis, urinary fistula, urinary incontinence, and urinary tract obstruction were recorded. Late GI toxicity with rectal hemorrhage was also noted.

**Table 3:** Toxicities after intensity-modulated radiation therapy.

Grade	Urinary			GI		
	Age <80	Age ≥80	P	Age <80	Age ≥80	P
***Acute***						
0, n (%)	221 (58.2)	115 (57.2)	0.860	224 (58.9)	130 (64.7)	0.182
1, n (%)	142 (37.4)	81 (40.3)	0.530	137 (36.1)	61 (30.3)	0.198
2, n (%)	17 (4.5)	5 (2.5)	0.263	18 (4.7)	9 (4.5)	1.000
3, n (%)	0	0	—	1 (0.3)	1 (0.5)	1.000
4, n (%)	0	0	—	0	0	—
5, n (%)	0	0	—	0	0	—
≥2, n (%)	17 (4.5)	5 (2.5)	0.263	19 (5.0)	10 (5.0)	1.000
≥3, n (%)	0 (0)	0 (0)	—	1 (0.3)	1 (0.5)	1.000
***Late***						
0, n (%)	337 (88.7)	185 (91.5)	0.248	261 (68.7)	135 (67.2)	0.209
1, n (%)	15 (3.9)	8 (4.0)	1.000	44 (11.6)	25 (12.4)	0.788
2, n (%)	20 (5.3)	7 (3.5)	0.410	56 (14.7)	32 (15.9)	0.716
3, n (%)	8 (2.1)	2 (1.0)	0.506	18 (4.7)	9 (4.5)	1.000
4, n (%)	0	0	—	1 (0.3)	0	1.000
5, n (%)	0	0	—	0	0	—
≥2, n (%)	28 (7.4)	9 (4.5)	0.212	75 (19.7)	41 (20.4)	0.913
≥3, n (%)	8 (2.1)	2 (1.0)	0.506	19 (5)	9 (4.5)	0.842

Kaplan-Meier survival analyses with log-rank tests of the actuarial probability of late grade 3 or higher urinary (p = 0.397) and GI (p = 0.966) toxicities showed no significant differences across the age strata. The 5-year incidence rates of grade 3 or higher urinary toxicity in patients younger than 80 years of age and 80 years of age or older were 3.1% and 0%, respectively. The actuarial 5-year GI toxicity rates for these groups were 6.8% and 5.1%, respectively. Late urinary and GI toxicity rates for the two age groups were statistically similar. Univariate and multivariate Cox regression analyses of late grade 3 or higher GI toxicity (Supplement Table 1) revealed mean dose to the rectum as the only significant risk factor of ≥grade 3 gastrointestinal toxicity.

## Discussion

This study found no significant differences in BFFS, DMFS, and DSS rates in prostate cancer patients aged 80 or older after definitive RT, compared with those under age of 80. The two age groups did not differ with respect to treatment-related toxicities. Beyond the well-known prognostic factors of tumor stage, Gleason score, and initial PSA, we found that latency before RT is a risk factor for biochemical failure after RT. Mean rectal dose was the only risk factor for late GI toxicity identified in this study.

Two prior studies found old age to be a poor prognostic factor after radical prostatectomy^11,12^. For RT, one study found advanced age to be an independent risk factor for prostate cancer-specific survival^13^. However, none of these studies took latency before definitive treatment into account, although treatment delay has been reported to be an independent predictor of time to PSA failure in high-risk prostate cancer^21^.

Latency to treatment may be longer in older patients. Older patients have been found previously to be less likely to receive treatment according to established guidelines^4,22^. In a study comparing monitoring and definitive therapy of 545 actively monitored men, 291 (53.4%) eventually required definitive treatments by the end of the study, with a follow-up duration of 10 years^23^. Hence, many older patients in previous studies who had a poor prognosis might have received conservative monitoring or ADT alone for many years before they received a definitive treatment.

This notion of possible prolonged latency in patients 80 years of age or older was confirmed in the present study. In treating an older patient population with RT following a uniform protocol, after a median follow-up of 66 months, no inferior prognosis with respect to treatment or toxicity was found in patients of 80 years of age or older compared with younger patients. Instead, prolonged latency before RT was shown to be a risk factor for biochemical failure.

The life expectancy of men age 80 or older in Taipei city in 2012 was 10.27 years. A life expectancy of greater than 10 years has been recommended by NCCN guidelines for deciding whether to arrange definitive RT in patients with low- and intermediate-risk prostate cancer^16^. International Society of Geriatric Oncology (SIOG) has recommended that healthy or fit older patients should have the same treatment options as younger patients^24^. In concordance with the recommendation, given that we did not observe any evidence suggestive of a difference in treatment outcomes or toxicities related to advanced age per se, we suggest that a robust man in his 80 s with prostate cancer should be treated with definitive RT similar to that given to younger patients without a prolonged latency.

The median age in the present cohort was the oldest among published case series of patients given IMRT for prostate cancer^25–29^. Although the follow-up durations are variable, a crude comparison of BFFS data from this series to those of prior series suggests that the presently observed BFFS data are comparable to those reported in the literature. We observed a higher BFFS than that in a prior dose escalation (86.4 Gy) study^28^, which may be attributable to a greater rate of ADT administration and pelvic irradiation.

Although advanced stage (T3 and T4) tumors were less prevalent in our older patient group than in our under 80 group, the proportions of patients in each risk group did not differ across the age strata. MRI is a more accurate diagnostic modality than CT^30^. Hence, the difference in tumor staging might be explained, at least in part, by the less frequent MRI staging in the older patients.

### Limitations

This study has several limitations that should be considered. First, the retrospective design of the study comes with inherent potential for biases. There may have been an increased risk of staging errors among cases staged without MRI. The compliance with oral ADT could not be ensured. Additionally, the use of traditional Chinese medicine, which is popular among patients with prostate cancer in Taiwan, was not recorded^31^. Only 8% patients received IGRT because of shortness in resources before. Finally, erectile function was not assessed.

## Conclusion

No differences in disease control or toxicity after definitive RT were observed between patients at least 80 years of age versus younger patients. Latency before RT was found to be a significant risk factor for biochemical failure and may explain the previous finding of inferior disease control in older patients.

## Electronic supplementary material

Supplementary Table
