# Supplementary material for: Definitive Radiotherapy for Older Patients with Prostate Cancer: Experience of a Medical Center in Taiwan
Source: Sci Rep. 2017 Oct 24;7:13880. doi: 10.1038/s41598-017-13119-3 (PMC5655670; doi:10.1038/s41598-017-13119-3)
Supplement: Supplementary file 1 — Supplementary Table [file 41598_2017_13119_MOESM1_ESM.doc]

Title:

Definitive Radiotherapy for Older Patients with Prostate Cancer: Experience of a Medical Center in Taiwan

Yuan-Hung Wu^1,2,3,4^, Wan-Chin Yang^1^, Yu-Wen Hu^1,2,3^, Chuen-Mei Hsieh^1^, Kai-Lin Yang^4,5,6^, I-Chun Lai^1,2^, Chen-Xiong Hsu^4,7^, Ti-Hao Wang^1,8^, Tzu-Yu Lai^1^, Kuan-Ting Chen^1^, Yu-Mei Kang^1^, Yu-Ming Liu^1,2^

1. Division of Radiation Oncology, Department of Oncology, Taipei Veterans General Hospital, Taipei, Taiwan

2. School of Medicine, National Yang-Ming University, Taipei, Taiwan

3. Institute of Public Health, National Yang-Ming University, Taipei, Taiwan

4. Department of Biomedical Imaging and Radiological Sciences, National Yang-Ming University, Taipei, Taiwan

5. School of Medicine, Fu Jen Catholic University, New Taipei City, Taiwan

6. Department of Radiation Therapy and Oncology, Shin Kong Wu Ho-Su Memorial Hospital, Taipei, Taiwan

7. Department of Radiation Oncology, Far Eastern Memorial Hospital, New Taipei City, Taiwan

8. Department of Radiation Oncology, China Medical University Hospital, Taichung, Taiwan

Corresponding author: Yu-Ming Liu M.D., Department of Oncology, Taipei Veterans General Hospital, No. 201 Sec.2 Shih-Pai Road, Taipei, Taiwan, 112

Email: [ymliug@gmail.com](mailto:ymliug@gmail.com)

Supplement Table 1: Cox proportional hazard model predicting ≥ grade 3 late gastrointestinal toxicity.

| **Risk factor** | **Univariate** | | **Multivariate** | |
| --- | --- | --- | --- | --- |
|  | **HR (95% CI)** | **P** | **HR (95% CI)** | **P** |
| Age at RT ≥ 80 | 0.984 (0.460–2.103) | 0.966 | 1.001 (0.464–2.160) | 0.997 |
| DM | 1.494 (0.684–3.263) | 0.314 | 1.588 (0.721–3.499) | 0.251 |
| Aspirin | 1.364 (0.663–2.809) | 0.399 | 1.311 (0.632–2.719) | 0.468 |
| Hemorrhoid | 1.932 (0.884–4.221) | 0.099 | 1.839 (0.839–4.035) | 0.128 |
| Rectal mean dose (Gy) | 1.195 (1.060–1.347) | 0.004* | 1.232 (1.081–1.404) | 0.002* |
| IGRT | 0.045 (0–111.5) | 0.437 | Not evaluable | 0.973 |
| Pelvic irradiation | 0.685 (0.332–1.413) | 0.306 | 0.560 (0.260–1.206) | 0.139 |

RT, radiotherapy; IGRT, image-guided RT
